# A Multicenter Randomized Controlled Prospective Study to Assess Efficacy of Laparoscopic Electrochemotherapy in the Treatment of Locally Advanced Pancreatic Cancer

**DOI:** 10.3390/jcm10174011

**Published:** 2021-09-05

**Authors:** Francesco Izzo, Vincenza Granata, Roberta Fusco, Valeria D’Alessio, Antonella Petrillo, Secondo Lastoria, Mauro Piccirillo, Vittorio Albino, Andrea Belli, Guglielmo Nasti, Antonio Avallone, Renato Patrone, Francesca Grassi, Maddalena Leongito, Raffaele Palaia

**Affiliations:** 1 Hepatobiliary Surgical Oncology Unit, Istituto Nazionale Tumori IRCCS Fondazione Pascale-IRCCS di Napoli, 80131 Naples, Italy; f.izzo@istitutotumori.na.it (F.I.); mauro.piccirillo@istitutotumori.na.it (M.P.); v.albino@istitutotumori.na.it (V.A.); a.belli@istitutotumori.na.it (A.B.); maddalena.leongito@istitutotumori.na.it (M.L.); r.palaia@istitutotumori.na.it (R.P.); 2 Radiodiodiagnostic Unit, Istituto Nazionale Tumori IRCCS Fondazione Pascale-IRCCS di Napoli, 80131 Naples, Italy; a.petrillo@istitutotumori.na.it; 3 IGEA SpA Medical Division—Oncology, Via Casarea 65, Casalnuovo di Napoli, 80013 Naples, Italy; r.fusco@igeamedical.com (R.F.); v.dalessio@igeamedical.com (V.D.); 4 Nuclear Medicine Unit, Istituto Nazionale Tumori IRCCS Fondazione Pascale-IRCCS di Napoli, 80131 Naples, Italy; s.lastoria@istitutotumori.na.it; 5 Abdominal Oncology Unit, Istituto Nazionale Tumori IRCCS Fondazione Pascale-IRCCS di Napoli, 80131 Naples, Italy; g.nasti@istitutotumori.na.it (G.N.); a.avallone@istitutotumori.na.it (A.A.); 6 PhD ICHT, University of Naples Federico II, 80131 Naples, Italy; dott.patrone@gmail.com; 7 Radiodiodiagnostic Unit, Università degli Studi della Campania Luigi Vanvitelli, 80128 Naples, Italy; francescagrassi1996@gmail.com; 8 Italian Society of Medical and Interventional Radiology (SIRM), SIRM Foundation, 20122 Milan, Italy

**Keywords:** electroporation, laparoscopic electrochemotherapy, locally advanced pancreatic

## Abstract

Background: Eighty percent of patients with pancreatic adenocarcinoma present a locally advanced or metastatic disease at diagnosis and are not eligible for surgery if not with palliative intent. In cases of locally advanced disease (LAPC), the combination of chemo and radiotherapy is the only therapeutic option and correlates with a median survival of 15 months (10 months without treatment), with partial remission of disease in 50% of cases. The feasibility and safety of Electrochemotherapy (ECT) have been demonstrated in the treatment of deep tumors. Aim: The aim of the study is to evaluate the efficacy of electrochemotherapy (ECT) followed by conventional systemic treatment compared to the only conventional systemic treatment in LAPC in terms of objective response and overall survival. Patients and Methods: This study is a phase IIb prospective multicenter randomized controlled trial with two arms. The study will include 90 patients: 45 in the control group and 45 in the experimental group. Patients with LAPC in the control arm will receive conventional chemotherapy (FOLFOXIRI). Patients with LAPC in the experimental arm will be subjected to Electrochemotherapy and subsequently to FOLFOXIRI. The objective response at 30, 90, and 180 days from treatment will be based on the computed tomography (CT), magnetic resonance (MR), and positron emission tomography/CT response (PET/CT). The objective long-term treatment response will be evaluated with the modified response evaluation criteria in solid tumors (m-RECIST) criteria, which will take into account the difference in vascularization, determined by the images obtained by CT and MR of the tumor treated before and after ECT. Conclusions: Not resectable liver metastasis, pancreatic tumors, and locally advanced renal carcinomas can be treated with laparoscopic electrodes. ECT could represent an effective therapeutic option for patients not eligible for surgery susceptible to be managed only with palliative therapies.

## 1. Introduction

Electroporation (EP) is a well-known methodology that allows a poorly or non-permeable molecule to pass through the cell membrane and reach the cytoplasm, thanks to the application of a short and intense electric field that determines an increase in permeability of the cell membrane. The combination of cytotoxic drug and EP is called electrochemotherapy (ECT).

The effectiveness of ECT with bleomycin has been in several cutaneous and subcutaneous tumors such as melanoma and chest wall breast cancer recurrence or for the treatment of squamous cell carcinoma of the head and neck [1,2,3,4,5,6,7,8,9,10,11,12,13]. Recently, the benefits of the ECT were also highlighted on deep solid tumors such as the liver and pancreas both in preclinical and clinical studies [14,15,16,17,18,19,20,21,22,23,24].

The only curative treatment is surgery; however, many patients have locally advanced or metastatic disease at diagnosis, and systemic chemotherapy is usually the main treatment [14]. The median survival of patients with metastatic disease treated with FOLFIRINOX therapy is only 3 mo [14]. FOLFIRINOX or modified FOLFIRINOX and gemcitabine/albumin-bound nab-paclitaxel remain the first-line treatment regimens, and for patients with BRCA1/2 and PALB2 mutations, FOLFIRINOX or modified FOLFIRINOX and gemcitabine/cisplatin are a second option [14]. Despite the recent introduction of novel chemotherapeutic schemes, these treatments still correlate with inadequate survival and significant systemic complications [14]. In our experience, by inserting a cytoreductive treatment in the multimodality approach of locally advanced disease, it is possible to obtain encouraging results both in terms of feasibility, safety, efficacy, and overall survival [14,15,22,23].

The use of ECT in deep cancer, e.g., liver and pancreas, currently requires a laparotomy surgical approach and limits its applicability due to the risks associated with open surgery. In our study, a new generation of electrodes suitable to treat tumors with ECT with a minimally invasive approach, i.e., laparoscopic surgery, will be used.

## 2. Trial Design

This is a phase IIb prospective multicenter randomized controlled trial with two arms. The study will include 90 patients: 45 in the control group and 45 in the experimental group.

### 2.1. Objectives

#### 2.1.1. Primary Endpoint

Increase evaluation of objective response rate in the experimental arm compared to the control arm.

#### 2.1.2. Secondary Endpoints

(a) To evaluate the effect of ECT on disease progression-free time and survival;
(b) To evaluate the impact of ECT on quality of life with particular attention to the effect on pain reduction;
(c) To evaluate the ECT toxicity;
(d) To evaluate by morphological and functional MRI parameters the conversion from locally advanced disease to resectable disease. The conversion rate will be calculated for each arm.

### 2.2. Subject’s Selection

The eligibility of the patients will be assessed by the investigators. Patient eligible will be informed about the study, and in case of consent to participate, he/her will sign the informed consent. Inclusion and exclusion criteria are summarized in Table 1.

### 2.3. Control Group

Patients with LAPC in the control arm will receive conventional chemotherapy (FOLFOXIRI).

FOLFOXIRI treatment is performed as follow: irinotecan 165 mg/m^2^ in 90-min iv infusion, oxaliplatin 85 mg/m^2^ in 120-min iv infusion (in a double path with folinic acid), folinic acid 200 mg/m^2^ in iv infusion of 120 min with oxaliplatin), 5-Fluorouracil 3200 mg/m^2^ iv in continuous 48 h infusion with an elastomeric pump. The treatment will be repeated every 14 days for a maximum of 12 cycles.

### 2.4. Experimental Group

Patients with LAPC in the experimental arm will be subjected to electrochemotherapy and subsequently to chemotherapy (FOLFOXIRI).

ECT will be performed following the standard operating procedures [3] via the laparoscopic approach.

Patients will receive 15,000 IU BLM/m^2^ intravenously. After 8 min, the ECT of the lesion will be performed using the CLINIPORATOR™ (IGEA S.p.A., Carpi, Italy) with the insertion of a new flexible, expandable electrode for laparoscopy treatment with CE certification. The procedure will be completed within 40 min. The treatment will be carried out under ultrasound guidance, and a preoperative planning tool using the PULSAR software (IGEA S.p.A., Carpi, Italy) will be performed when multiple insertions of single needles will be required. The use of the software allows estimation of the electric field required in the region of interest, calculating an optimized treatment in terms of electrode configuration (number and position), voltage, and distance for each couple of electrodes within or around the predefined area segmented by the user.

After the ECT procedure has been accomplished, using the new probe, the carbon dioxide is released out of the abdomen through the slits, and then these sites are closed with sutures or staples or covered with glue-like bandages and steri-strips.

**Figure 1:**
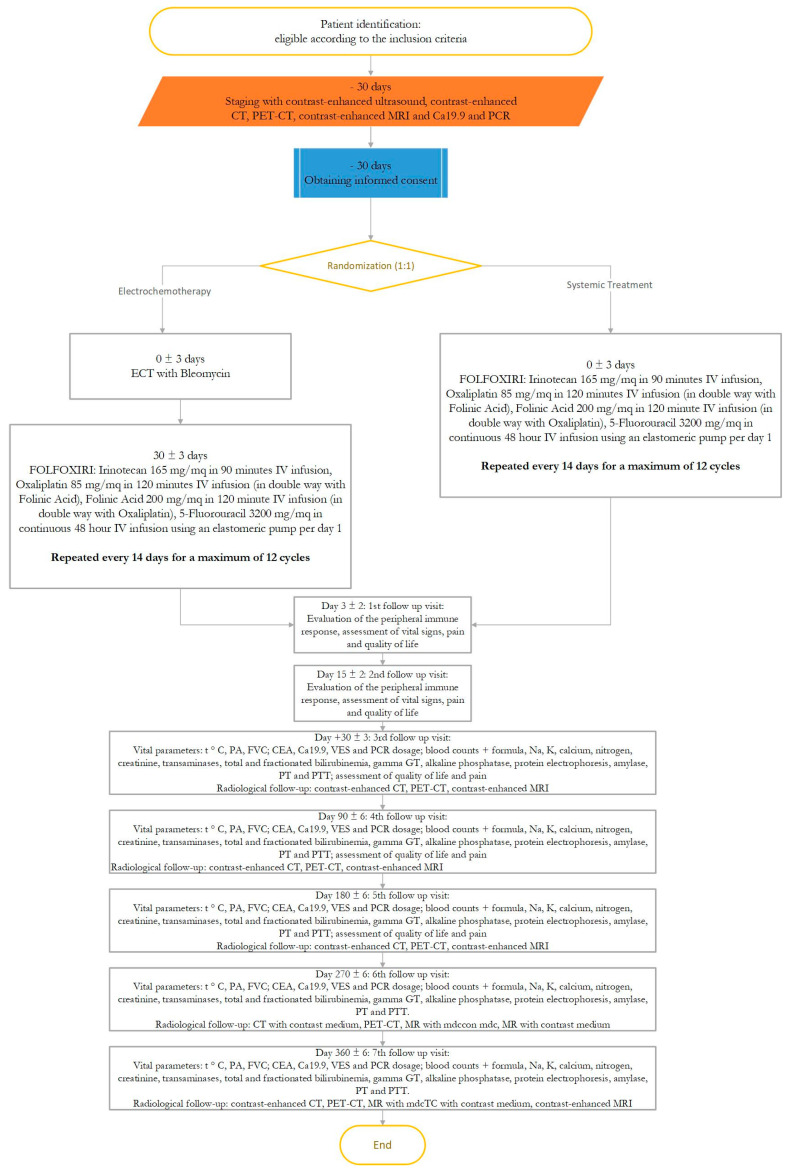
Trial design flowchart.

The patients in the experimental group will receive FOLFOXIRI treatment at day +30 from ECT as indicated above. The FOLFOXIRI treatment will be repeated every 14 days for a maximum of 12 cycles. The flowchart of the trial design is shown in Figure 1.

### 2.5. Endpoints Evaluation Criteria

The efficacy of the treatment will be evaluated in terms of the objective response rate of the treated lesion in the experimental arm compared to the control arm.

The objective response, at 30, 90, and 180 days from treatment, will be assessed on computed tomography (CT), magnetic resonance, and positron emission tomography/CT (PET-CT) performed using morphological criteria (response evaluation criteria in solid tumors (RECIST) version 1.1 [25]), modified RECIST (m-RECIST [26]), CHOI criteria [27], and PET response criteria in solid tumors (PERCIST) criteria [28]) and functional parameters extracted by MR sequences. The evaluation phase of the MR images will include analysis by morphological dimensional criteria (RECIST 1.1) and parameters extracted from the post-processing of DCE-MRI and DWI data. The post-processing phase of DCE-MRI images will provide both semi-quantitative analysis of intensity-time curves (TIC) and quantitative analysis based on pharmacokinetic models [29,30]. The variables calculated in the semi-quantitative analysis will be: the enhancement of healthy parenchyma in early and late phase; slope and intercept of wash-in phase (absorption phase of cm) and wash-out phase (excretion of cm); area under curve. The variables calculated in the quantitative analysis will be: Ktrans transfer constant from plasma to extravascular extracellular space; Kep constant rate from extra extracellular extravascular to plasma space; ve fraction of volume occupied by extra extracellular extravascular space; vp fraction of volume occupied by plasma. The post-processing of DWI images will include the calculation of the apparent diffusion coefficient (ADC), which integrates the diffusion and perfusion effects, and the intravoxel incoherent motion (IVIM) parameters: D, the pure diffusion coefficient related to the macroscopic motion of water molecules; f, the perfusion fraction; D *, the pseudo diffusion coefficient bound to the microscopic blood movements in the capillaries.

The objective long-term treatment response (270 and 360 days from the procedure) will be evaluated with the m-RECIST criteria, which will take into account the difference in size and vascularization of the tumor before and after ECT.

Morphological and functional MRI (MR functional parameters extracted by dynamic contrast-enhanced magnetic resonance imaging (DCE-MRI) and diffusion-weighted imaging (DWI)) will be used to evaluate the conversion from locally advanced disease to resectable disease at 270 and 360 days from the procedure.

### 2.6. Secondary Endpoint of the Study

Analysis of disease progression-free time and survival will be made. During the follow-up period, duration of complete tumor regression, recurrence appearance, and its response to salvage treatment, causes of death will be documented. Overall and progression-free survival will be assessed by Kaplan–Meier analysis.

Disease recurrence rates will be compared between two groups. Median and range will be recorded. Quality of life (QoL), pain, and satisfaction questionnaires scores will be collected for each patient. The EORTC QLQ-C30 questionnaire for QoL evaluation will be will administered to the patients at the time of inclusion and 30, 90, 180 days after treatment [24].

No specific toxicities are expected, and no adverse events are predicted except muscle contraction during the treatment and pain thereafter. However, each adverse event will be collected. NCI-CTCAE classification (version 5.0) will be used to the intensity of adverse events evaluation.

The duration of the study will be 48 with 12 months follow-up for each patient.

The follow-up visit will be performed 1, 2, 4, 6, 8, 10, and 12 months after the treatment fixing the cut-off point for tumor response evaluation at 2 months. CT, MRI, or PET-CT will be performed at 2, 6, and 12 months from treatment. The visits scheme is shown in Table 2.

### 2.7. Description of Study Procedures

#### 2.7.1. Visit 1

Before undergoing any procedure, all patients must sign informed consent. They will undergo the same enrollment/baseline visit (Visit 1) procedures not more than 30 calendar days before Visit 2.

Patients will be interviewed, and if they meet the inclusion and exclusion criteria, they will be randomized 1:1 to ECT or standard chemotherapy.

The following vital parameters will be evaluated: Tmax °C, blood pressure (PA), forced vital capacity (FVC); dosage carcinoembryonic antigen (CEA), carbohydrate antigen 19.9 (Ca 19.9), velocity erythrocyte sedimentation (VES), and protein C reactive (PCR); blood count + formula, sodium (Na), potassium (K), calcium (Ca), nitrogen, creatinine, transaminases, total and fractionated bilirubinemia, gamma-glutamyl transferas (y-GT), alkaline phosphatase, protein electrophoresis, amylase, prothrombin time (PT), partial thromboplastin time (PTT), and international normalized ratio (INR).

Radiologic evaluation will be performed to measure the size, morphological and functional data of the tumor by means of conventional instrumental investigations: MR with contrast medium (cm), CT with cm, and PET-CT.

Oncology history of the patients, previous chemotherapy, and response to prior therapy, concomitant treatments, and each event for each visit will be collected by the investigator.

The patient will be subjected to a physical examination that includes weight, height, and vital signs.

Complete blood count (CBC) and coagulation profiles will be recorded no more than 7 calendar days before Visit 2. Patients with an absolute neutrophil count below 1000/mL, platelet count below 70,000/mL, and/or INR above 1.5 will be excluded.

Pain will be measured using the 100 mm anchored visual analog scale with 0 being “No Pain” and 100 mm being “Pain as bad as it could possibly be”.

Quality of life, as measured by the European Organization for Research and Treatment of Cancer (EORTC) QLQ-C30) questionnaire [24], will be completed at Visit 1, 3, 4, 5, and 6. The ECOG (Eastern Cooperative Oncology Group) performance status rates the condition of the patient as reported in the work of [31]. Patients who are grade 0, 1, or 2 are eligible.

All patients will be subjected to pre-anesthesia assessment, and patients found not eligible for general anesthesia will be excluded.

Pregnancy will be established prior to enrollment by beta-human chorionic gonadotropin (beta-HCG) assay on urine (pregnancy test or urinary beta-HCG with a highly sensitive test as per CTFG guidelines) or blood (plasma beta-HCG).

#### 2.7.2. Visit 2

On the established day, the patients will undergo ECT treatment (experimental group) or FOLFOXIRI (control group).

For each patient, the evaluation of vital parameters, peripheral blood collected in EDTA, QoL, and pain will be monitored.

Immunological parameters will be evaluated to identify possible diagnostic and predictive markers of disease. In this phase will be analyzed two groups of immunological parameters: circulating cell populations and serum cytokines. Cellular subset will be evaluated: CD4+ and CD8+ T cells will be subdivided according to the expression of CD45RA and CCR7 in: naïve (CD45RA+/CCR7+), central memory (CD45RA-/CCR7+), effector memory (CD45RA-/CCR7-), and terminal effector (CD45RA+/CCR7). The expression of PD1 and TIM-3, LAG-3, CTLA-4, granzyme B, and perforin markers will be evaluated for each CD8 exhaustion population and cytotoxic CD8; Tregs cells are identified as a percentage of CD4+CD25+ CD127 low FOXP3+ cells. The percentage of CD4+CD25hiFOXP3+ CD127 low positive for cytotoxic T-lymphocyte-associated protein 4 (CTLA-4), inducible T-cell COStimulator (ICOS), ectonucleoside triphosphate diphosphohydrolase-1 (ENTPD1), CD45RA, programmed cell death protein 1 (PD-1), and chemokine receptor type 4 (CXCR4) will be determined; myeloid and plasmocytoid dendritic cells (mDC and pDC) are identified as LIN2-/HLA-DR+ cells and then subdivided in myeloid DC (mDC: CD11c+/CD16-) and plasmocytoid DC (pDC: CD123+/CD11c-). The expression of CD83 and CD86 markers will be evaluated for each DC population.

The plasma concentration of several metabolites such as cytokines, interleukins, and chemokines able to modulate the immune response are analyzed: interleukin (IL)-6, stromal cells-derived factor (SDF)-1, IL-1β, tumor necrosis factor (TNF)-α, interferon (IFN)-γ, vascular endothelial growth factor (VEGF), and transforming-growth factor (TGF)-β, nonhistone chromatin protein high-mobility group box 1 (HMGB1) and calreticulin (CRT).

The ECT procedure will be performed as follow: administration of bleomycin for i.v. infusion and treatment delivering with CLINIPORATOR™ and appropriate electrode probe CE marked.

#### 2.7.3. Visit 3 to Visit 9 (Follow-Up Visit)

The patients will be subjected to the following procedures: assessment of vital parameters; radiologic examination (contrast media-enhanced CT and (MRI), PET-CT); QoL and pain evaluation (EORTC QLQ-C30 questionnaire and VAS scale); and peripheral blood collected in ethylenediaminetetraacetic acid (EDTA) and evaluations as for Visit 2.

### 2.8. Description of Sample Size Calculation

Sample size estimation has been performed by using the superiority hypothesis in two independent parallel sample proportions [32]. Primary endpoint objective tumor response (OR = CR + PR) between two groups. According to literature data, studies report a response rate in the control arm in the range of 25–40%.

Assuming an approximately 30% increase in the response rate provided by ECT compared to standard systemic treatment, the minimum number of patients needed for the study is 45 per arm.

Group sample sizes of 45 in Group 1 (experimental group) and 45 in Group 2 (control group) achieve 80% power to detect a difference between the group proportions of 0.3000. The Group 2 proportion is 0.400. The Group 1 proportion is assumed to be 0.4500 under the null hypothesis and 0.7000 under the alternative hypothesis. The test statistic used is the one-sided *t*-test. The significance level of the test was targeted at 0.0500.

### 2.9. Statistical Analysis

Descriptive analysis of group participants at study entry. Mean and standard deviation will be presented for continuous variables if normally distributed: if not normally distributed, median and interquartile range will be exhibited. The difference between follow-up visit and baseline will be calculated and will be tested by 2-sided Student *t*-test or by Mann–Whitney test. ANOVA test or Kruskal–Wallis test will be used to test the null hypothesis by adjusting for proper covariates [33]. For categorical variables, the chi-square test will be used. Differences will be considered significant at a *p*-value < 0.05. The objective response will be reported as a categorical variable and marked as a response rate.

Quality of life and pain assessment will be evaluated as continuous variables.

The difference in VAS pain score between each follow-up visit and the baseline will be computed will be tested using the Mann–Whitney test.

Kaplan–Meier analysis will be used for overall and progression-free survival evaluation. Disease recurrence rates and percentages will be compared between the two groups.

Analysis of conversion rate from locally advanced disease to resectable disease, chi-square test will be performed to assess differences statistically significant among groups.

Interim analyses are fixed after the first step of the study at 12 months, and the expansion of the trial at others centers will be evaluated based on enrollment percentage. Conclusive results analysis will be performed at the end of the study. No safety concerns have arisen in earlier studies with the device and bleomycin in combination to suggest the need for early stopping for safety reasons. Any planned changes to the statistical plan as implied by the statistical considerations summary above will be discussed in advance.

## 3. Discussions

Adenocarcinoma of the pancreas is among the most aggressive forms of cancer [34]. Currently, chemotherapy and/or radiotherapy are the standard therapies in locally advanced and metastatic disease. First-line treatment regimens remain FOLFIRINOX or modified FOLFIRINOX [34,35,36,37]. Although new chemotherapy regimens are used, these treatments are associated with inadequate survival and are not devoid of systemic complications. Additionally, only one-third of patients are responsive to chemotherapy [33,36].

Although there are no randomized study results indicating an additional role of ablative treatments to chemotherapy alone, nor studies completed comparing the various ablative modalities, patients with persistent locally advanced disease, who are in suitable clinical condition (WHO Performance Status 0–1), and response evaluation criteria in solid tumors (RECIST) stable disease after 2–4 months chemotherapy can be treated by local ablation therapies. The increased interest in these treatments is related to the fact that they seem to favor the systemic antitumor response, and therefore, their combination with immunotherapy could improve disease control. However, ablative treatments should only be employed in locally growing pancreatic cancers and used as consolidation treatments in a multimodal approach [38,39,40,41,42,43,44,45,46].

Recently, the benefits of the ECT were observed and documented on deep solid tumors such as the liver and pancreas both in preclinical and clinical studies [17,18,19,20,21,22,23]. Compared to other thermal ablation techniques, ECT is free of thermal effects allowing complete treatment of localized lesions close to vessels and bile ducts. Electrochemotherapy should be employed as a stage in a multimodal treatment that is completed with the combination of chemotherapy and/or radiotherapy.

### 3.1. Preclinical Experiences

ECT in pancreatic cancer has been investigated by Jaroszeski et al. [21] in a preclinical trial using a hamster animal model in which tumoral cells were injected directly into the pancreas. The combination of EP with intratumoral bleomycin was able to induce a 25% response. Safety of the procedure was demonstrated in vivo by Girelli et al. [20] in the normal pancreas of the rabbits suggesting that electroporation could be a valid alternative for the local control of non-resectable pancreatic cancer since it does not damage the normal pancreatic parenchyma.

### 3.2. Clinical Experience

The feasibility, safety, and efficacy results of ECT by means of the CLINIPORATORTM VITAE generator model of a prospective clinical phase I/II study were published by Granata et al. [22,23] and reported encouraging results. No acute (intraoperative) and/or postoperative serious adverse events related to ECT were observed; no clinically significant electrocardiographic, hemodynamic, or serum biologic changes were noted. No clinically relevant elevation of amylase or lipase levels was observed in any patient, and no bleeding or damage to surrounding viscera occurred.

Functional imaging based on MR and PET scans was demonstrated to be more suitable to evaluate ECT response in patients with locally advanced pancreatic adenocarcinoma than CT imaging. According to the authors, ECT of locally advanced pancreatic adenocarcinoma is a feasible, safe, and effective treatment modality [14,22,23,47,48].

The aim of the study is to evaluate the efficacy of electrochemotherapy followed by conventional systemic treatment compared to the only systemic treatment in LAPC in terms of objective response.

Treatment with ECT of deep-seated lesions, either percutaneously or during laparoscopic/endoscopic procedures, is at its early stages, but this approach looks promising. Laparoscopic/endoscopic ECT of solid organs is a novel, minimally invasive treatment modality and potentially very effective [49].

The advantages of laparoscopic surgery compared to open surgery, are numerous such as faster recovery, hospital stay, and hospital costs reduction. Laparoscopic cancer surgery is associated with better outcomes in terms of reduced surgical complications and perioperative morbidity [50,51,52,53,54]. The new prototype of electrodes used in this study is suitable for laparoscopic/c treatments and for use in combination with laparoscopic ports and endoluminal optical instrumentation. The electrode configurations allow a gradual increase in the ablated area in consecutive steps, as shown in our preclinical study on pigs [55]. Treatment of anatomical areas excluded until now is possible thanks to miniaturization of the electrode and to the divergence of the needle. Laparoscopic electrodes can be used for not resectable liver metastasis, pancreatic tumors, and locally advanced renal carcinomas [56,57,58,59,60]. ECT could represent an effective therapeutic option for patients not eligible for surgery susceptible to be managed only with palliative therapies [56,57,58,59,60,61,62,63,64,65].

## Figures and Tables

**Table 1:** Inclusion and exclusion criteria.

Inclusion Criteria	Exclusion Criteria
Age ≥ 18 years	Age less than 18 years
Suitable mental health conditions	Absolute contraindication to surgery
Ability to sign a specific informed consent in order to be enrolled in the study	Visceral, bone, or diffuse metastases
Life expectancy in line with the follow-up indicated by the study	Presence of extrahepatic spread of the disease
Diagnosis of exocrine pancreatic cancer with histological confirmation	Clinically significant ascites
Preoperative Staging (CT and MRI) of locally advanced pancreatic cancer disease: stage III	Any serious and uncontrolled systemic illness
The subject is not eligible for the “gold-standard” treatment of surgical pancreatectomy and is eligible for a conventional systemic treatment (FOLFOXIRI)	Acute lung infection
	Symptoms of poor lung function by clinical examination and Pulmonary function tests (PFT)
	Noncorrectable severe coagulation disorders
	Contraindications at the assumption of bleomycin
	Previous adverse reactions to bleomycin
	Cumulative dose of ≥250 mg/m^2^ of bleomycin
	Pregnancy or lactation

Eligible patients will be randomly assigned (1:1) to the experimental group or control group.

**Table 2:** Visit scheme.

Visit		1	2		3	4	5	6	7	8	9
**Time**		Month −1 (up to 1 Month Prior the Treatment)	Month 0		Month 1 ± 1 Week	Month 2 ± 1 Week	Month 4 ± 1 Week	Month 6 ± 1 Week	Month 8 ± 1 Week	Month 10 ± 1 Week	Month 12 ± 1 Week
	**Visit Description**	Restaging, Enrollment, Randomization	Day of Treatment Control Group: Cetuximab+Platinum+5- Fluorouracil Experimental Group: Electrochemotherapy	Post-Treatment Evaluation (Discharge Day ± 1 Week)	Follow-Up Visit	Follow-Up Visit Cut-Off Time to Evaluate Treatment Response	Follow-Up Visit	Follow-Up Visit	Follow-Up Visit	Follow-Up Visit	Follow-Up Visit
**Type of Assessment**											
**Clinical evaluation**			**X**	**X**	**X**	**X**	**X**	**X**	**X**	**X**	**X**
**Duration of hospitalization**				**X**							
**CT, MRI or PET-CT**		**X**	**Only estimation of Lesion size**			**X**		**X**			**X**
**Identification of the target lesion**		**X**	**X**	**X**							
**Photographic documentation**		**X**	**X**		**X**	**X**	**X**	**X**	**X**	**X**	**X**
**EORTC QLQ-C30, EORTC QLQ-H&N35, EQ-5D-5L questionnaires**		**X**		**X**	**X**	**X**	**X**	**X**	**X**	**X**	**X**
**Pain evaluation with VAS score**		**X**		**X**	**X**	**X**	**X**	**X**	**X**	**X**	**X**
**Blood samples as per normal clinical practice**		**X**	**X**		**X**	**X**	**X**	**X**	**X**	**X**	**X**
**CD8 and CD16 dosage**			**X**		**X**	**X**	**X**	**X**			
**Recording of the drugs for pain control**				**X**		**X**	**X**	**X**	**X**	**X**	**X**
**Recording of concomitant treatment**					**X**	**X**	**X**	**X**	**X**	**X**	**X**
**ECOG status**		**X**			**X**	**X**	**X**	**X**	**X**	**X**	**X**
**Adverse Events/Complications**					**X**	**X**	**X**	**X**	**X**	**X**	**X**

## Data Availability

All data are reported in the manuscript.
